# Flagellum expression and swimming activity by the zoonotic pathogen *Escherichia albertii*

**DOI:** 10.1111/1758-2229.12818

**Published:** 2019-12-25

**Authors:** Koichi Murakami, Shinya Kimura, Osamu Nagafuchi, Tsuyoshi Sekizuka, Daisuke Onozuka, Fuminori Mizukoshi, Hiroyuki Tsukagoshi, Taisei Ishioka, Tetsuo Asai, Shinichiro Hirai, Manami Musashi, Motoi Suzuki, Makoto Ohnishi, Kazunori Oishi, Nobuhiro Saruki, Hirokazu Kimura, Sunao Iyoda, Makoto Kuroda, Shuji Fujimoto

**Affiliations:** ^1^ Infectious Disease Surveillance Center National Institute of Infectious Diseases Musashi‐Murayama Tokyo Japan; ^2^ Gunma Prefectural Institute of Public Health and Environmental Sciences Maebashi Gunma Japan; ^3^ Fukuoka Institute of Technology Fukuoka Fukuoka Japan; ^4^ Pathogen Genomics Center National Institute of Infectious Diseases Shinjuku Tokyo Japan; ^5^ Department of Preventive Medicine and Epidemiology National Cerebral and Cardiovascular Center Suita Osaka Japan; ^6^ Department of Microbiology Tochigi Prefectural Institute of Public Health and Environmental Science Utsunomiya Tochigi Japan; ^7^ Takasaki City Health Center Takasaki Gunma Japan; ^8^ The United Graduate School of Veterinary Sciences Gifu University Gifu Japan; ^9^ Aomori Prefectural Public Health and Environment Center Aomori Japan; ^10^ Department of Bacteriology I National Institute of Infectious Diseases Shinjuku Tokyo Japan; ^11^ Gunma Paz University Takasaki Gunma Japan; ^12^ Kyushu University Fukuoka Fukuoka Japan

## Abstract

Flagella are the well‐known structural appendages used by bacteria for motility. Although generally reported to be non‐motile, the enteropathogenic bacterial species *Escherichia albertii* produces flagella intermittently. We found that *E. albertii* expressed flagella under specific environmental conditions. After several generations (involving 4 to 12‐h incubations), six of the twelve strains we investigated displayed swimming motility in various aquatic environments, including pond water containing nutrients from pigeon droppings (10% suspension) as well as in 20 × −diluted tryptic soy broth. The most significant motility determinant was a temperature between 15 and 30 °C. At 20 °C in the 10% pigeon‐dropping suspension, microscopic observations revealed that some cells (1%–95% of six strains) showed swimming motility. Electron microscopy showed that the *E. albertii* cells expressed flagella. Lower concentrations of some substrates (including nutrients) may be of secondary importance for *E. albertii* flagella expression. Interestingly, the non‐motile strains (*n* = 6/12) contained pseudogenes corresponding to essential flagella structural proteins. After being released from its host into surface water, *E. albertii* may express flagella to move toward nutrient sources or new hosts.

## Introduction

The specific conditions under which flagella are expressed in the enteropathogenic bacterial species *Escherichia albertii* merit investigation. The structure and components of flagella, which are the tail‐like appendages used by bacteria for motility, have been extensively studied (Berg, 2003; Sowa and Berry, 2008). While approximately 70% of bacterial species express flagella (Aizawa, 2004), a few bacteria, such as *Yersinia enterocolitica* (Kapatral *et al*., 1996), intermittently express flagella in response to environmental cues. Flagella‐based motility, namely swimming (including tumbling) and swarming (Kearns, 2010), allows bacterial cells to move toward more optimal growth environments and nutrient sources. However, like most *Shigella* species, the zoonotic diarrheal pathogen *E. albertii* was not thought to produce flagella (Nataro *et al*., 2007). *E. albertii*, a pathogen first described in 2003 (Huys *et al*., 2003), is generally believed to be non‐motile based on the observations made under standard laboratory conditions (Nataro *et al*., 2007). But in 2015, Ooka *et al*. reported that this emerging zoonotic pathogen harbours intact flagellum operons within its genome (Ooka *et al*., 2015). Therefore, the aim of the current study was to examine whether flagellum expression occurs in *E. albertii* in surface water after being released from host animals such as birds.

## Results and discussion

### *Microscopy‐based examination of swimming motility*

**Figure 1:**
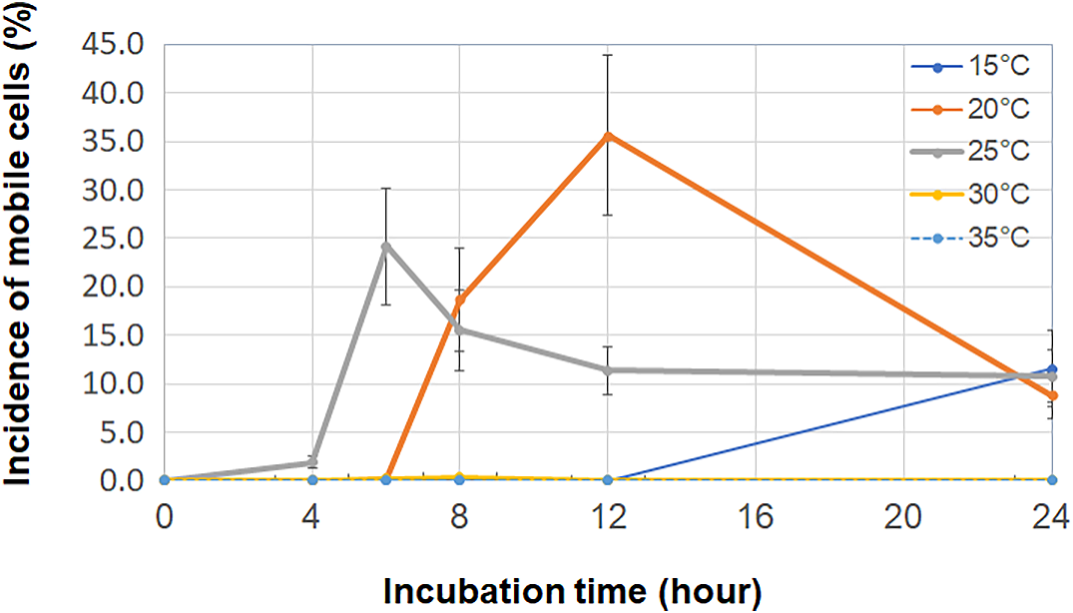
The average motility frequency among cells from six motile strains out of twelve *Escherichia albertii* strains tested. After 4 to 12‐h incubations, six of the twelve strains were investigated displayed swimming motility in 10% pigeon‐dropping suspension. Motility frequency was determined for 200 *E. albertii* cells from each of motile (*n* = 6) and non‐motile (*n* = 6) strains (altogether, 2,400 cells were counted) in 10% pigeon‐dropping suspension with the dropping pool from Site A and the surface water from Pond A at different temperatures over several incubation periods. The motile (*n* = 6) and non‐motile (*n* = 6) strains tested are listed in Supporting Information Table S1. All strains were randomly selected from our collection (*n* = ~200) and are available upon request. Cells were observed by light microscopy. A significant (*p* < 0.001) percentage of cells were motile at 20 or 25 °C, with increased motility observed at several growth temperatures after 12 h of incubation (*p* < 0.001). Six strains exhibited swimming (including tumbling) motility (ranging from 1% to 95% of 200 cells), whereas the remaining six strains were non‐motile. Bars show the standard errors obtained from three or four replicates. The proportion (%) of 200 cells showing swimming (including tumbling) motility was then determined at 0, 3, 6, 8, 12, and 24 h post‐inoculation by examining the cells by phase‐contrast microscopy (BX‐51; Olympus, Co., Tokyo, Japan) using microscope slides and coverslips (1.8 × 1.8 cm; Matsunami Glass Ind., Ltd., Kishiwada, Japan). A linear regression model was used to investigate associations between temperature, incubation period and bacterial cell counts in the statistical analysis.

**Figure 2:**
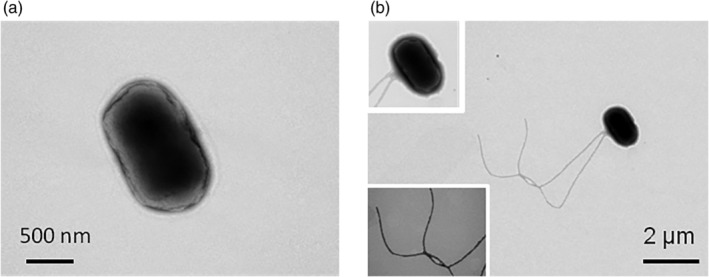
Electron microscopic images of *Escherichia albertii*. Images of *E. albertii* strain 3908 cells following culture in (A) 1× tryptic soy broth at 35 °C or (B) 10% pigeon‐dropping pond water suspension at 20 °C. Flagella were expressed under the conditions described in (B). Pond water samples were collected from two ponds in the Tokyo Metropolitan Area (Pond A) and in Fukuoka Prefecture (Pond B). Samples were collected in sterilized bottles on day 3 of three consecutive sunny days in 2018. Water samples were filtered using 0.22‐μm pore‐size membranes (Merck KGaA, Darmstadt, Germany) and stored at −80 °C until use. Fresh pigeon‐dropping pools were collected from parks and temples in Kyoto (Site A), Kanagawa (Site B) and Tokyo (Site C) as previously described (Murakami *et al*., 2014). Additional fresh pigeon droppings were donated by a pigeon racing association (Tokyo, Japan) (Site D). Droppings collected from each geographical area were pooled and then stored at −80 °C. The dropping pool from Site A and the surface water from Pond A were mainly used in this study. To prepare the 10% pigeon‐dropping suspension, pigeon droppings were suspended in nine volumes of pond water and then filtrated through a 0.22‐μm filter. TSB was prepared using milliQ water. Dilutions (1.25‐, 1.67‐, 2.5‐, 5‐, and 20‐fold) of the medium were then prepared in milliQ water. Following incubation of the tested strains in TSB at 42 °C (to reflect the body temperature of pigeons) for 15 ± 1 h, a 1‐ml aliquot of each culture was centrifuged (22,000 × *g*, 1 min), and the resulting cell pellet resuspended in 1 ml of either the appropriate test medium or milliQ water. A 10‐μl aliquot of the suspension was then inoculated into 1 ml of 10% pigeon‐dropping suspension or diluted or undiluted TSB.

Motility assays confirmed that some of the tested *E. albertii* strains were motile. Of the 12 strains (Supporting Information Table S1) initially examined, six displayed swimming motility in pond water (from Pond A) where 10% pigeon droppings were suspended (dropping pool from Site A). Motility was observed at temperatures ranging from 15 to 30 °C following 6–24 h of incubation (Fig. 1, Supporting Information Movie S1) under a light microscope. Following incubation for 12 h at 20 °C, some cells (1%–95% of six strains) were seen to be swimming (Fig. 1). Electron microscopy‐based examination of the *E. albertii* cells revealed the presence of flagella (Fig. 2). No difference in the number of strains showing swimming motility was recorded among the different combinations of pigeon droppings (dropping pools from Sites A, B, C and D) and pond water samples (from Ponds A and B) used to make the pigeon‐dropping suspensions.

### *Next‐generation sequencing*

Using the whole genome sequences from the 12 strains, the six swimming‐negative strains were found to harbour several flagella structural protein‐encoding pseudogenes (*flgG*, *flgI*, *flgK*, *flhA*, *fliD* and *fliF*) (Supporting Information Table S1).

### *Soft agar‐based examination of swimming motility*

**Figure 3:**
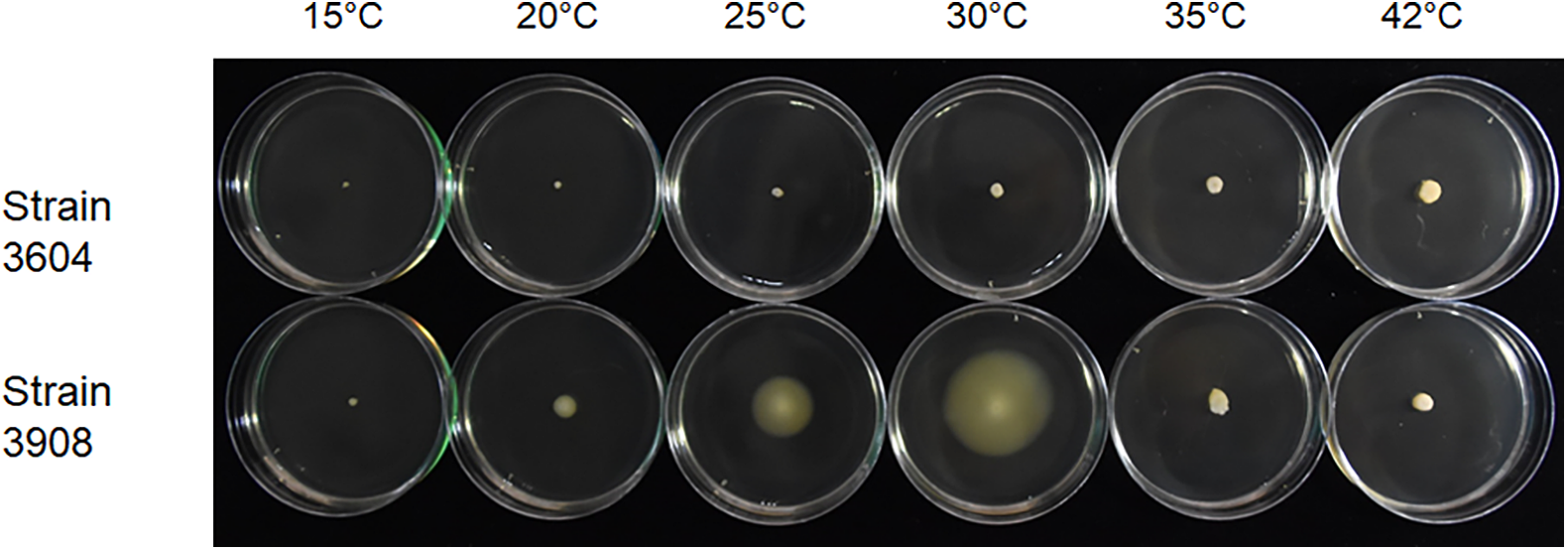
Swimming activity of *Escherichia albertii* strains. Strains 3604 and 3908 were cultured on 10% pigeon‐dropping suspension agar (0.25% agar, w/v) and incubated for 3 days at 30 °C. Strain 3604 contains the *flgG* pseudogene and was therefore non‐motile.

In soft ager tests on swimming motility conducted using 10% pigeon‐dropping suspension agar (made from dropping pool from Site A and surface water from Pond A), the six motile *E. albertii* strains swam at temperatures ranging from 15 to 30 °C after a 3‐day incubation period, although the greatest swimming diameter was observed at 30 °C (Fig. 3). The six motile strains also swam in agar made from various dilutions of tryptic soy broth (TSB; Becton, Dickinson, and Company, Franklin Lakes, NJ, USA) at 30 °C, with the swimming motility observed in 1.7 × −20 × −diluted agar‐supplemented TSB medium (Supporting Information Fig. S2). However, at 35 °C, no swimming motility was observed in either the pigeon‐dropping suspension or in the TSB agar media (Fig. 3). Following these initial experiments, an additional 85 *E. albertii* strains were screened for motility in 10% pigeon‐dropping suspension agar at 30 °C for 3 days. Of these, 40 (47.1%) strains displayed swimming motility.

### *Growth assay*

The optimum temperature (35 °C) for bacterial propagation in the 10% pigeon‐dropping suspension was determined by conducting growth assays at various incubation temperatures (Supporting Information Fig. S1). No remarkable differences were observed among the 12 strains.

### *Survival assay*

All 12 *E. albertii* strains survived for 4 weeks at 20 °C in surface water from Pond A (mean cell count decreased from 8.0 × 10^8^ ± 1.1 × 10^8^ colony‐forming units (CFU)/ml to 1.4 × 10^6^ ± 1.5 × 10^5^ CFU/ml) or in surface water from Pond B (mean cell count decreased from 8.2 × 10^8^ ± 1.2 × 10^8^ CFU/ml to 9.6 × 10^6^ ± 1.4 × 10^6^ CFU/ml).

Altogether, these findings provide important information regarding the cues governing *E. albertii* motility. Temperatures lower than the body temperatures of endothermic animals may be the most significant determining factor for the promotion of flagella expression. Lower concentrations of some substrates (including nutrients) might also promote motility based on the observed increase in swimming motility (Supporting Information Figs. S2 and S3) of *E. albertii* cells cultured on or in diluted TSB agar or TSB media, respectively, with osmolality, and/or viscosity also likely to be involved (Chaban *et al*., 2015). Despite not being the most important factor, nutrition is still a significant driver of *E. albertii* flagella expression because bacterial growth and propagation (generational change) is necessary during incubation for flagella to be expressed (Aizawa and Kubori, 1998). Figure S1 (Supporting Information) shows that no obvious differences were recorded among the swimming and non‐swimming strains, suggesting that the latter strains were mature enough for flagella expression. As shown in Supporting Information Table S2, the concentration of nutrients in the growth medium, including the pond water, played a significant role in flagella expression. Many chemical components were present in much lower concentrations in the 10% pigeon‐dropping medium compared with the 1× TSB medium (Supporting Information Table S2). Further examination may identify the key cues contributing to bacterial motility using a more advanced experimental method, because the centrifugal speed used for cell preparation (22,000 × g, 1 min) in the present study (Fig. 1, legend) could be improved.

*E. albertii* can find a new host via its swimming motility. Our results confirm that *E. albertii* can survive for several weeks in low‐nutrient surface water conditions. Surface water, including pond water, frequently reaches temperatures as high as 20 °C in many countries (Sharma *et al*., 2015). After being released from its host (e.g., in bird droppings) into surface water, and after several generations, *E. albertii* can express flagella to move toward nutrient sources or new hosts (Supporting Information Fig. S4).

The intermittent flagellum expression by *E. albertii* may help to augment our understanding of pathogen evolution. The *E. albertii* chromosome is relatively large (~4.6 Mb) and contains numerous protein‐coding sequences (~4,200) (Ooka *et al*., 2015), suggesting that it is a free‐living or facultative pathogen rather than an obligate pathogen or symbiont (Moran, 2002). Because its main transmission route is via environmental water sources, survival tools such as flagella would be beneficial for *E. albertii*. Identifying the pseudogenes corresponding to flagella structural proteins in half of the strains we examined herein may mark the evolution of *E. albertii* from a facultative to an obligate pathogen (Moran, 2002) (i.e., a host‐adapted or host‐restricted pathogen (Thomson *et al*., 2008)), which is usually characterized by genome reduction and virulence factor loss (Merhej *et al*., 2013). Moreover, the presence of several pseudogenes in six of the twelve strains suggests that several strains of *E. albertii* have adapted to a specific host mammal or bird in accordance with where they are transmitted directly between hosts; such a lifestyle would not require swimming motility. Such strains have lost flagella, a known functional pathogenicity factor (Haiko and Westerlund‐Wikström, 2013). Generally, waterborne transmission favours evolution toward high virulence (Ewald, 1991), whereas virulence reduction is important for vertical transmission (Yamamura, 1993) of pathogens to maximize the basic reproduction ratio (Lion and Metz, 2018). Therefore, some *E. albertii* strains may have evolved for vertical transmission.

In conclusion, some strains of *E. albertii* display swimming motility after several generations in aquatic environments containing nutrient sources such as pigeon droppings.

### *Ethics statement*

The study protocols were approved by the National Institute of Infectious Diseases for Public Health Ethics Committees (No. 576). The pigeon dropping sampling locations are not privately owned and are not protected in any way. The field studies did not involve endangered or protected species. At no point did the researchers come into physical contact with the pigeons.

The funders had no role in the study design, data collection and analysis, decision to publish or preparation of the manuscript.

## Supporting information


**Fig. S1** Growth of motile (*n* = 6) and non‐motile (*n* = 6) *Escherichia albertii* strains in 10% pigeon‐dropping suspension. Growth was examined at four different temperatures and viable cell counts were determined by plate‐based colony counts on nutrient agar. Bars indicate the standard error of the means of three or four replicates.

**Fig. S2** Swimming activity of *Escherichia albertii. E. albertii* swimming on diluted tryptic soy broth (TSB) agar (0.25%) (dilutions indicated), undiluted TSB agar (0.25%), and basal (water only) agar (0.25%) plates (6‐cm diameter) following incubation at 30°C for 3 days. Strain 3604 lacks *flgG* and was therefore non‐motile.

**Fig. S3** Average motility frequency among *Escherichia albertii* cells in dilute tryptic soy broth (TSB). Proportion (%) of motile cells among 200 *E. albertii* cells from each of 12 different strains (altogether, 2,400 cells were counted) following growth in dilute TSB (dilutions indicated) at 20°C. Bars show the standard error of the means of three replicates.

**Fig. S4** Survival advantage conveyed by the expression of flagella by *Escherichia albertii* in the environment. *E. albertii* expresses flagella to move towards more optimal environments or towards new hosts after being released from the original host.

**Table S1** *Escherichia* albertii strains tested and accession numbers of DNA sequences deposited in the DDBJ/GenBank/EMBL databases.

**Table S2** Constituents of media used to confirm motility of *Escherichia albertii* (mg/l).

**Table S3** Methods used to determine the chemical characteristics of pond water and culture media.

**Movie S1** Swimming motility of *Escherichia albertii* strain 3908. *E. albertii* 3908 was incubated in 10% pigeon‐dropping suspension (dropping pool from Site A and pond water A) at 20°C.

**Appendix S1:** Supporting Information
