# Supplementary figures and images for: Flagellum expression and swimming activity by the zoonotic pathogen Escherichia albertii
Source: Environ Microbiol Rep. 2019 Dec 25;12(1):92–6. doi: 10.1111/1758-2229.12818 (PMC7003939; doi:10.1111/1758-2229.12818)

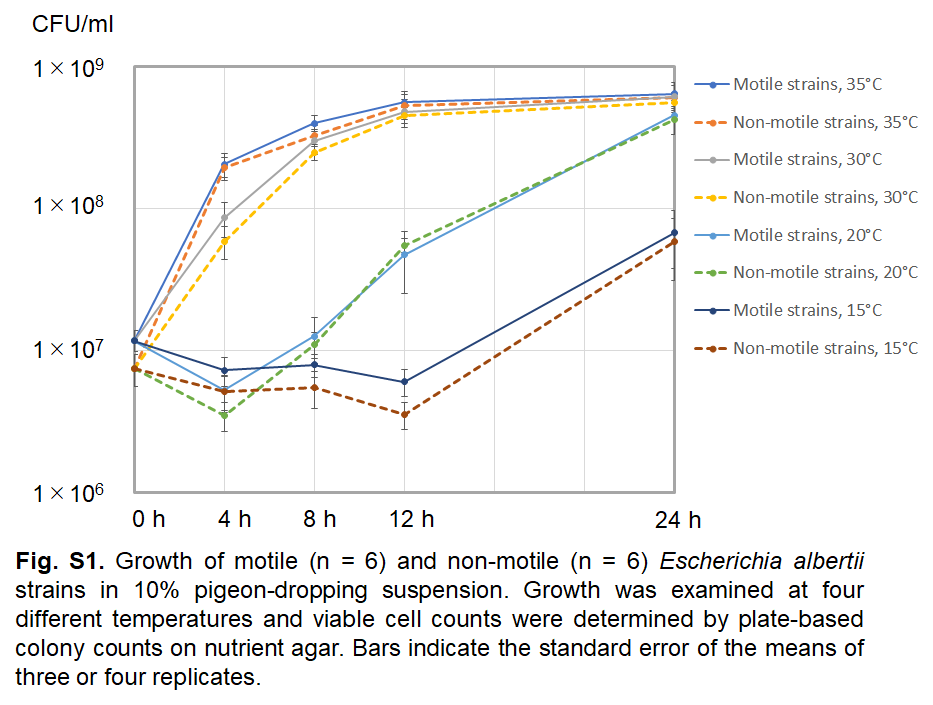

Supplement: Supplementary file 1 — Fig. S1 Growth of motile (n = 6) and non‐motile (n = 6) Escherichia albertii strains in 10% pigeon‐dropping suspension. Growth was examined at four different temperatures and viable cell counts were determined by plate‐based colony counts on nutrient agar. Bars indicate the standard error of the means of three or four replicates. [file EMI4-12-92-s001.tif]

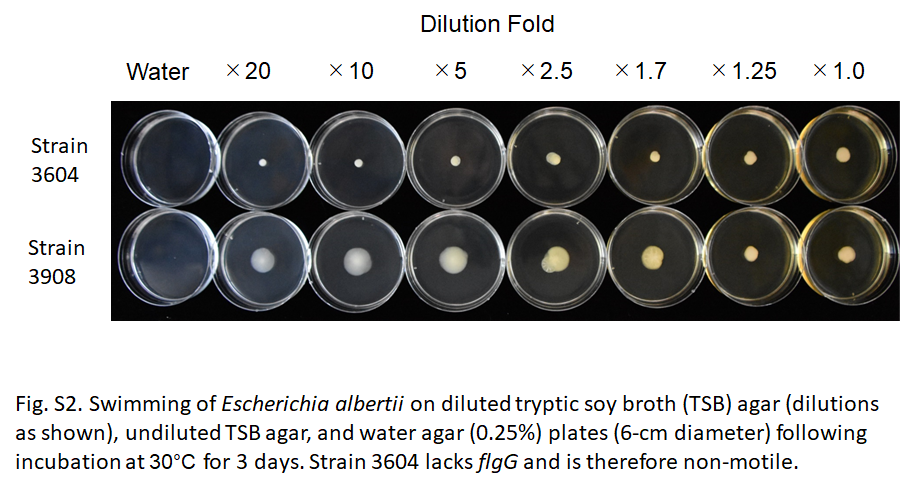

Supplement: Supplementary file 2 — Fig. S2 Swimming activity of Escherichia albertii. E. albertii swimming on diluted tryptic soy broth (TSB) agar (0.25%) (dilutions indicated), undiluted TSB agar (0.25%), and basal (water only) agar (0.25%) plates (6‐cm diameter) following incubation at 30°C for 3 days. Strain 3604 lacks flgG and was therefore non‐motile. [file EMI4-12-92-s002.tif]

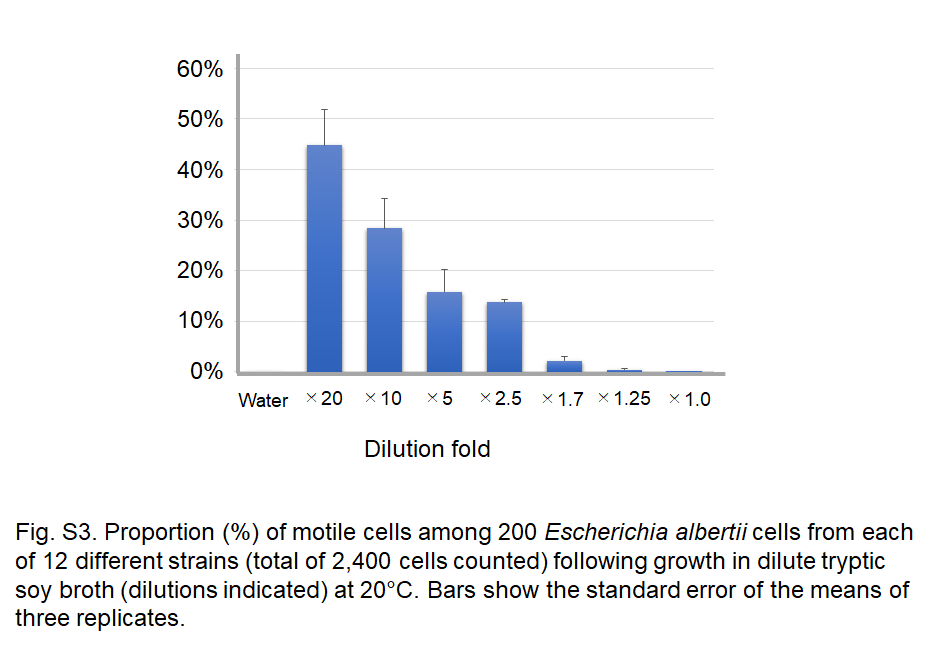

Supplement: Supplementary file 3 — Fig. S3 Average motility frequency among Escherichia albertii cells in dilute tryptic soy broth (TSB). Proportion (%) of motile cells among 200 E. albertii cells from each of 12 different strains (altogether, 2,400 cells were counted) following growth in dilute TSB (dilutions indicated) at 20°C. Bars show the standard error of the means of three replicates. [file EMI4-12-92-s003.tif]

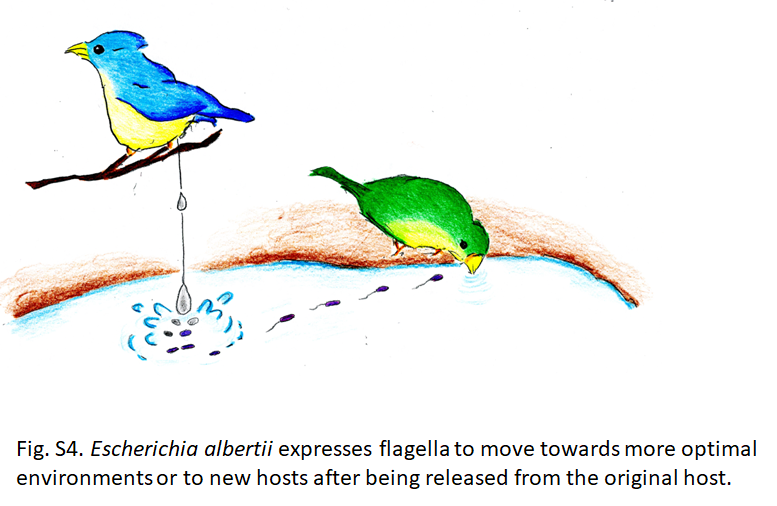

Supplement: Supplementary file 4 — Fig. S4 Survival advantage conveyed by the expression of flagella by Escherichia albertii in the environment. E. albertii expresses flagella to move towards more optimal environments or towards new hosts after being released from the original host. [file EMI4-12-92-s004.tif]
