# Supplementary material for: Flagellum expression and swimming activity by the zoonotic pathogen Escherichia albertii
Source: Environ Microbiol Rep. 2019 Dec 25;12(1):92–6. doi: 10.1111/1758-2229.12818 (PMC7003939; doi:10.1111/1758-2229.12818)
Supplement: Supplementary file 5 — Table S1 Escherichia albertii strains tested and accession numbers of DNA sequences deposited in the DDBJ/GenBank/EMBL databases. [file EMI4-12-92-s005.docx]

Table S1. *Escherichia albertii* strains tested and accession numbers of DNA sequences deposited in the DDBJ/GenBank/EMBL databases^a^

| Strain number |  | Strain name | Origin |  | Motility (swimming)^b^ |  | Pseudogenes of flagella structural genes^c^ |  | Genbank accession number |
| --- | --- | --- | --- | --- | --- | --- | --- | --- | --- |
| 3260 |  | ZAH-1-3 | Wild bird (pigeon) |  | Motile |  | Not found |  | BJCT01000001-BJCT01000202 |
| 3282 |  | ZAI-5-1 | Wild bird (pigeon) |  | Motile |  | Not found |  | BJCU01000001-BJCU01000043 |
| 3302 |  | ZAI-52-1 | Wild bird (pigeon) |  | Non motile |  | *flgK, fliF* |  | BJCV01000001-BJCV01000281 |
| 3333 |  | U350-1 | Wild bird (pale thrush) |  | Non motile |  | *flhA* |  | BJCW01000001-BJCW01000121 |
| 3359 |  | AZK-6-2 | Wild bird (pigeon) |  | Non motile |  | *flgK, fliF* |  | BJCX01000001-BJCX01000228 |
| 3406 |  | AZN-8-1 | Wild bird (pigeon) |  | Non motile |  | *fliD* |  | BJCY01000001-BJCY01000137 |
| 3604 |  | Cyou_D3 | Wild bird in a harbor (uncertain) |  | Non motile |  | *flgG* |  | BJCZ01000001-BJCZ01000113 |
| 3908 |  | Gyo_GY2 | Wild bird (starling) |  | Motile |  | Not found |  | BJDA01000001-BJDA01000079 |
| 3915 |  | Gifu_53 | Wild bird (great cormorant) |  | Motile |  | Not found |  | BJDB01000001-BJDB01000166 |
| 3964 |  | Mkr3964 | Human (patient) |  | Non motile |  | *flgI* |  | BJDC01000001-BJDC01000241 |
| 3965 |  | Mkr3965 | Human (patient) |  | Motile |  | Not found |  | BJDD01000001-BJDD01000233 |
| 3972 |  | Esc18014 | Human (patient) |  | Motile |  | Not found |  | BJDE01000001-BJDE01000069 |

^a^DNA sequences were obtained by next-generation sequencing

^b^Based on results from all assays conducted in this study; All of the six motile strains showed swimming.

^c^*flgG*: gene coding for distal rod protein; *flgI:* gene coding for P ring protein; *flgK*: gene coding for hook-filament junction protein; *flhA*: gene coding for flagellin export apparatus protein; *fliD*: gene coding for filament cap protein; *fliF*: gene coding for MS ring protein
