# Supplementary material for: Flagellum expression and swimming activity by the zoonotic pathogen Escherichia albertii
Source: Environ Microbiol Rep. 2019 Dec 25;12(1):92–6. doi: 10.1111/1758-2229.12818 (PMC7003939; doi:10.1111/1758-2229.12818)
Supplement: Supplementary file 6 — Table S2 Constituents of media used to confirm motility of Escherichia albertii (mg/l). [file EMI4-12-92-s006.docx]

Table S2. Constituents of media used to confirm motility of *Escherichia albertii* (mg/l^a^)

| Characteristics and chemical composition of constituents | TSB^b^ | Dissolved pigeon droppings (10%) in pond water | Pond water | Comparing value (10% dissolved pigeon droppings/TSB) | Comparing value (Pond water/TSB) |
| --- | --- | --- | --- | --- | --- |
| pH | 7.1 | 6.9 | 7.7 |  |  |
| Ammonia nitrogen | 61 | 31 | <0.01 | 51% | 0.8% |
| Calcium ions | 9.2 | 65 | 21 | 707% | 76.8% |
| Chloride ions | 3000 | 66 | 6.7 | 2% | 0.0% |
| Magnesium ions | 18 | 63 | 3.1 | 350% | 19.4% |
| Nitrate nitrogen | <0.5 | 1.6 | <0.01 |  |  |
| Nitrite nitrogen | 3.5 | 0.52 | ＜0.01 | 15% | 4.2% |
| Phosphate/phosphorus | 530 | 69 | <0.05 | 13% | 0.0% |
| Potassium ions | 1400 | 190 | 2.0 | 14% | 0.0% |
| Sodium ions | 4000 | 65 | 8.0 | 2% | 0.0% |
| Sulfate ions | 74 | 20 | 3.9 | 27% | 0.4% |
| Aluminum | 0.23 | 0.050 | 0.039 | 22% | 94.5% |
| Iron | 0.54 | 0.12 | 0.021 | 22% | 41.2% |
| Boron | 0.21 | 0.12 | 0.012 | 57% | 272.1% |
| Copper | 0.010 | 0.21 | 0.00082 | 2100% | 210000.0% |
| Manganese | 0.028 | 0.12 | 0.00063 | 429% | 15306.1% |
| Molybdenum | 0.031 | 0.0005 | 0.0003 | 2% | 52.0% |
| Zinc | 0.71 | 0.20 | 0.0013 | 28% | 39.7% |
| Dissolved organic carbon | 8600 | 1200 | 4.2 | 14% | 0.0% |
| Total organic carbon | 9300 | 1200 | 4.2 | 13% | 0.0% |
| Dissolved total nitrogen | 2300 | 360 | 0.34 | 16% | 0.0% |
| Total nitrogen | 2300 | 360 | 0.35 | 16% | 0.0% |
| Dissolved total phosphorus | 600 | 68 | <0.03 | 11% |  |
| Total phosphorus | 600 | 68 | <0.03 | 11% |  |

^a^Except for pH

^b^Tryptic soy broth (Becton, Dickinson, and Company, Franklin Lakes, NJ, USA) made with MilliQ water
