# Supplementary material for: Flagellum expression and swimming activity by the zoonotic pathogen Escherichia albertii
Source: Environ Microbiol Rep. 2019 Dec 25;12(1):92–6. doi: 10.1111/1758-2229.12818 (PMC7003939; doi:10.1111/1758-2229.12818)
Supplement: Supplementary file 7 — Table S3 Methods used to determine the chemical characteristics of pond water and culture media. [file EMI4-12-92-s007.docx]

Table S3. Methods used to determine chemical characteristics of pond water and culture media

| Detection target |  | Method | Detector | Underlying law and notification in Japan |
| --- | --- | --- | --- | --- |
| pH |  | Glass electrode | F 73 (Kayaba, Tokyo, Japan) |  |
| Ammonia nitrogen |  | Ion chromatography following filtration through 0.45-µm pore-size filter (Sllhh25ns; Merck, Darmstadt, Germany) | Dionex ICS-1100 (Thermo Fisher Scientific, Waltham, MA, USA) | JIS K 0102 42.5 |
| Calcium ions |  |  |  | JIS K 0102 50.4 |
| Chloride ions |  |  |  | JIS K 0102 35.3 |
| Magnesium ions |  |  |  | JIS K 0102 51.4 |
| Nitrate nitrogen |  |  |  | JIS K 0102 43.2.5 |
| Nitrite nitrogen |  |  |  | JIS K 0102 43..1.2 |
| Phosphate/phosphorus |  |  |  | JIS K 0102 46.1.3 |
| Potassium ions |  |  |  | JIS K 0102 49.3 |
| Sodium ions |  |  |  | JIS K 0102 48.3 |
| Sulfate ions |  |  |  | JIS^a^ K 0102 41.3 |
| Aluminium |  | Inductively-coupled plasma mass spectrometry following acidification of the samples with 1% HNO_3_ | 7800 ICP-MS (Agilent Technologies, Inc., Santa Clara, CA, USA) | Notification no. 261, Ministry of Health, Labor, and Welfare, Japan (2003) |
| Iron |  |  |  |  |
| Boron |  |  |  | JIS K 0102 47.4 |
| Copper |  |  |  | Notification No. 261, Ministry of Health, Labor, and Welfare, Japan (2003) |
| Manganese |  |  |  |  |
| Molybdenum |  |  |  | JIS K 0102 68.3 |
| Zinc |  |  |  | JIS K 0102 53.4 |
| Dissolved organic carbon^b^ |  | Non-dispersive infrared gas detection with high temperature oxidation combustion | TOC-V-CSN (Shimadzu Co., Kyoto, Japan) | JIS K 0102 22.2 |
| Total organic carbon |  |  |  | JIS K 0102 22.2 |
| Dissolved total nitrogen^b^ |  | Chemiluminescence detection |  | JIS K 0102 45.5 |
| Total nitrogen |  |  |  | JIS K 0102 45.5 |
| Dissolved total phosphorus^c^ |  | Colourimetry with potassium peroxodisulfate decomposition following thermal cracking at 120°C for 30 min | UV-1800 (Shimadzu Co., Kyoto, Japan) | JIS K 0102 46.3.1 |
| Total phosphorus |  |  |  | JIS K 0102 46.3.1 |

^a^JIS, Japanese Industrial Standard

^b^Samples were initially filtered using a 0.45-µm pore-size filter (Sllhh25ns; Merck, Darmstadt, Germany)

^c^Samples were initially filtered using a No. 5C (Toyo Roshi Kaisha, Ltd, Tokyo, Japan)
