# Supplementary material for: Flagellum expression and swimming activity by the zoonotic pathogen Escherichia albertii
Source: Environ Microbiol Rep. 2019 Dec 25;12(1):92–6. doi: 10.1111/1758-2229.12818 (PMC7003939; doi:10.1111/1758-2229.12818)
Supplement: Supplementary file 9 — Appendix S1: Supporting Information [file EMI4-12-92-s009.docx]

**Supplementary Information**

**Flagellum expression and swimming activity by the zoonotic pathogen *Escherichia albertii***

**Experimental Procedures**

***Strains tested***

The *E. albertii* strains (*n* = 12) tested in the current study are listed in Supporting Information Table S1. All strains were randomly selected from our collection (*n* = ~200) and are available upon request.

***Pond water***

Water samples were collected from two ponds in the Tokyo Metropolitan Area (Pond A) and in Fukuoka Prefecture (Pond B). Samples were collected in sterilized bottles on day 3 of three consecutive sunny days in 2018. Water samples were filtered using 0.22-µm pore-size membranes (Merck KGaA, Darmstadt, Germany) and stored at −80°C until use.

***Pigeon droppings***

Fresh pigeon droppings were collected from parks and temples in Kyoto (Site A), Kanagawa (Site B), and Tokyo (Site C) as previously described (Murakami et al., 2014). Additional fresh pigeon droppings were donated by a pigeon racing association (Tokyo, Japan) (Site D). Droppings collected from each geographical area were pooled and then stored at −80°C. The dropping pool from Site A was mainly used in this study.

***Broth***

To prepare 10% pigeon dropping suspension, pigeon droppings were suspended in nine volumes of pond water and then filtrated through a 0.22-μm filter. TSB was prepared using milliQ water. Dilutions (1.25-, 1.67-, 2.5-, 5-, and 20-fold) of the medium were then prepared in milliQ water.

***Motility assay and movie***

Following incubation of the tested strains in TSB at 42°C (to reflect the body temperature of pigeons) for 15 ± 1 h, a 1-ml aliquot of each culture was centrifuged (22,000 × *g*, 1 min) and the resulting cell pellet resuspended in 1 ml of either the appropriate test medium or milliQ water. A 10-μl aliquot of the suspension was then inoculated into 1 ml of 10% pigeon dropping suspension or diluted or undiluted TSB. The proportion (%) of 200 cells showing swimming (including tumbling) motility was then determined at 0, 3, 6, 8, 12, and 24 h post-inoculation by examining the cells by phase-contrast microscopy (BX-51; Olympus, Co., Tokyo, Japan) using microscope slides and coverslips (1.8 × 1.8 cm; Matsunami Glass Ind., Ltd., Kishiwada, Japan). Microscopic movies were recorded using a DP27-C-2 camera (Olympus, Co.).

***Plate assay for assessing swimming motility***

Preculture was conducted as described for the motility assay. Swimming was examined on the various TSB agars and on 10% pigeon-dropping suspension agar in 6-cm diameter Petri dishes. TSB dilution agars consisted of 1.25-, 1.67-, 2.5-, 5-, 10-, and 20-fold dilutions of TSB medium supplemented with 0.25% agar (w/v, Bacto Agar; Becton, Dickinson, and Company). The 10% pigeon dropping suspension agar consisted of 20% pigeon-dropping suspension mixed with molten 0.5% agar (final agar concentration, 0.25% (w/v)).

***Electron microscopy***

Aliquots of bacterial culture were fixed with an equal volume of 4% paraformaldehyde and 4% glutaraldehyde (w/v) in 0.1 M phosphate buffer (pH 7.4). The samples were then adsorbed to formvar film-coated copper grids and stained with 2% phosphotungstic acid solution (w/v, pH 7.0) for a few seconds. The grids were then observed under a transmission electron microscope (JEM-1400Plus; Jeol Ltd., Akishima, Japan) at an acceleration voltage of 100 kV. Digital images (3296 × 2472 pixels) were captured using a charge-coupled device camera (EM-14830RUBY2; Jeol Ltd.).

***Survival in pond water***

Preculture was conducted as described in the motility assay using surface water Ponds A and B. Cell suspensions were incubated at 20°C for 4 w. Plate-based colony counts to determine the viable cell numbers in each suspension were performed for both pre- and post-incubation using nutrient agar (Eiken Chemical Co., Tokyo, Japan).

***Propagation of the bacterial strains***

To determine the optimal growth temperature for the strains, all 12 strains were cultivated at different temperatures for several different time periods. Plate-based colony counts were conducted at the end of the experimental periods using nutrient agar.

***Next-generation sequencing (NGS)***

Total genomic DNA extracted from the 12 strains was analyzed by NGS as described previously (Shigemura et al., 2018). The resulting contigs containing the genes encoding flagella structural proteins were validated using BLASTN homology searches against a database constructed from complete nucleotide sequences obtained from the NCBI database (Supporting Information Table S1).

***Statistical analysis***

A linear regression model was used to investigate associations between the temperature, incubation period and bacterial cell counts in the statistical analysis (Fig. 1).

***Chemical analysis***

The methods used to determine the chemical characteristics of the pond water and culture media are shown in Supporting Information Table S3.

**References**

Murakami, K., Etoh, Y., Ichihara, S., Maeda, E., Takenaka, S., Horikawa, K. et al. (2014) Isolation and characteristics of Shiga toxin 2f-producing *Escherichia coli* among pigeons in Kyushu, Japan. *PLoS One* **9**: e86076.

Shigemura, H., Matsui, M., Sekizuka, T., Onozuka, D., Noda, T., Yamashita, A. et al. (2018) Decrease in the prevalence of extended-spectrum cephalosporin-resistant *Salmonella* following cessation of ceftiofur use by the Japanese poultry industry. *Int J Food Microbiol* **274**: 45-51.
